# A Mouse Tracking Study of the Perception of English Affricate Onsets in Native Mandarin Learners

**DOI:** 10.1007/s10936-025-10195-9

**Published:** 2026-01-21

**Authors:** Yizhou Wang, Rikke L. Bundgaard-Nielsen

**Affiliations:** 1 School of Languages and Linguistics, The University of Melbourne, Parkville, VIC 3052 Australia; 2 MARCS Institute for Brain, Behaviour & Development, Penrith, NSW 2751 Australia

**Keywords:** Mouse-Tracking, Affricates, Perception, Identification, Discrimination

## Abstract

This paper reports on an experimental study investigating native Mandarin listeners’ perception of two English contrasts involving affricates, /dʒ/-/dɹ/, and /tʃ/-/tɹ/, using a combination of an AXB discrimination task and a Mouse Tracking (MT) identification task. Results show that Mandarin listeners experience substantial difficulty discriminating English affricates when such consonants precede an /u/ vowel, but find discrimination easy when preceding an /i/ vowel, revealing a pervasive effect of phonotactic constraints in Mandarin syllable phonology. The results also demonstrate that MT technology complements traditional psycholinguistic techniques like discrimination tasks and reveals additional information about nonnative listeners’ online processing of speech during the decision-making process. By comparing the AXB and MT identification results, we report both similarities and complementarities of the keystroke and mouse-clicking paradigms that should be taken into consideration of L2 perception research designs for tapping into different modes of speech processing. The findings of the present study thus contribute to both our understanding of the influence of native phonology on L2 speech perception, as well as psycholinguistic methodologies for L2 perception research.

## Introduction

It is well-established that acquiring second language (L2) phonology can be challenging, and theoretical accounts for such L2 difficulties have taken many forms, including the Perceptual Assimilation Model (PAM/PAM-L2) (Best, 1995; Best & Tyler, 2007), the Automatic Selective Perception model (ASP) (Strange, 2011; Strange & Shafer, 2008), the Speech Learning Model (SLM/SLM-revised) (Flege, 1995; Flege & Bohn, 2021), and the Second Language Linguistic Perception model (L2LP) (Escudero, 2009; van Leussen & Escudero, 2015). To date, most frameworks aim to account for the difficulties that arise from mismatches of various kinds between the L1 and L2 phonological inventories: A classic example is the difficulties experienced by Japanese learners of English with the /l/-/ɹ/ liquid contrast because Japanese only has one liquid phoneme while English has two (Cutler, 2012; MacKain et al., 1981; Strange & Dittmann, 1984). Some models, and in particular those with a gestural approach to phonology like PAM/PAM-L2 (Best, 1995; Best & Tyler, 2007), also highlight that L2 difficulties arise when the realisation of L2 target phones represents an unfamiliar coordination of the articulation events to what is required in the listener’s native language (e.g., Wang et al., 2023a, 2023b), whether this is on a segmental level or phonotactic level (Dupoux et al., 1999, 2011; Kilpatrick et al., 2019, 2020).

The present study has a dual motivation. Firstly, we explore native Mandarin listeners’ perception of English affricate onsets /tʃ dʒ/ and the stop-rhotic sequences /tɹ dɹ/, which together represent a very interesting case of articulatorily complex phones/sequences in which nonnative (English) stops have delayed and affricated releases, which may pose challenges to speakers of Mandarin (Lan, 2020a, 2020b). In contrast to English post-alveolar affricates /tʃ dʒ/, Mandarin has a different set of affricates: Laminal affricates /tɕ dʑ/ preceding high front segments, and apical affricates /tʃ̺ dʒ̺/, although the transcription symbols may differ across studies in the literature (Duanmu, 2007; Lee & Zee, 2003; Lin, 2007; for instance, /tʂʰ tʂ/). In a parallel fashion, English /tɹ dɹ/ may pose a challenge because Mandarin syllable phonotactics does not permit stop-rhotic or affricate-rhotic sequences. In fact, Mandarin phonology only permits a small number of diphone (CC) onset clusters, where the second consonant is restricted to a glide, i.e., /j, w, ɥ/. As a result, sequences like English /tɹ/ and /dɹ/ are therefore likely be perceived by listeners as phonotactically illicit and may even be perceptually “repaired” in some way.

Mandarin loanwords with an English origin do indeed suggest that /tɹ/ and /dɹ/ are sometimes “repaired” and adapted as affricate + /w/ sequences. We speculate that this is because English /ɹ/’s are typically characterised by some degree of lip-rounding, and such a secondary articulation feature can be exploited by Mandarin speakers for loan adaptation. For example, *Trump* is often adapted as *Chuanpu* /**tʃw**an.pu/ while *Charlie* is adapted as /**tʃ**a.li/. Importantly, these examples indicate that a contrast between /tʃ/ and /tɹ/ is maintained after adaptation via different cross-language mapping strategies (via exploiting the labial gesture in the rhotic), providing indirect evidence of differential perception. At the same time, /tɹ/ and /tʃ/ are likely to merge when the nucleus vowel is /u/ e.g., *Truman* is often adapted as /**tʃ**u.mən/ but not /**tʃw**u.mən/. As Mandarin does not have a contrast between /wu/ and /u/ (Duanmu, 2007, 2011), and this suggests that the perceptual exploitation of the labial gesture in rhotics, i.e., /tɹ/ → /tʃw/, becomes unavailable in the /u/ context due to a lack of native (Mandarin) contrast between vowels and glide-vowel sequences (e.g., the contrast between *east* and *yeast*). This repair is unlikely to be primarily due to the constraints in Mandarin Chinese orthography, as psycholinguistic account of loanword adaptation (Peperkamp, 2004, 2015) suggest that such adaptations indicate challenges in the nonnative listeners’ auditory perception of such L2 consonants, evidenced in loanword shapes. As a result, this psycholinguistic account will predict that Mandarin listeners should have difficulty in perceiving English /tʃ/-/tɹ/ and /dʒ/-/dɹ/ contrasts at the syllable onset position when the nucleus vowel is /u/, while they should have no difficulty when the nucleus vowel is not a high back vowel.

**Fig. 1:**
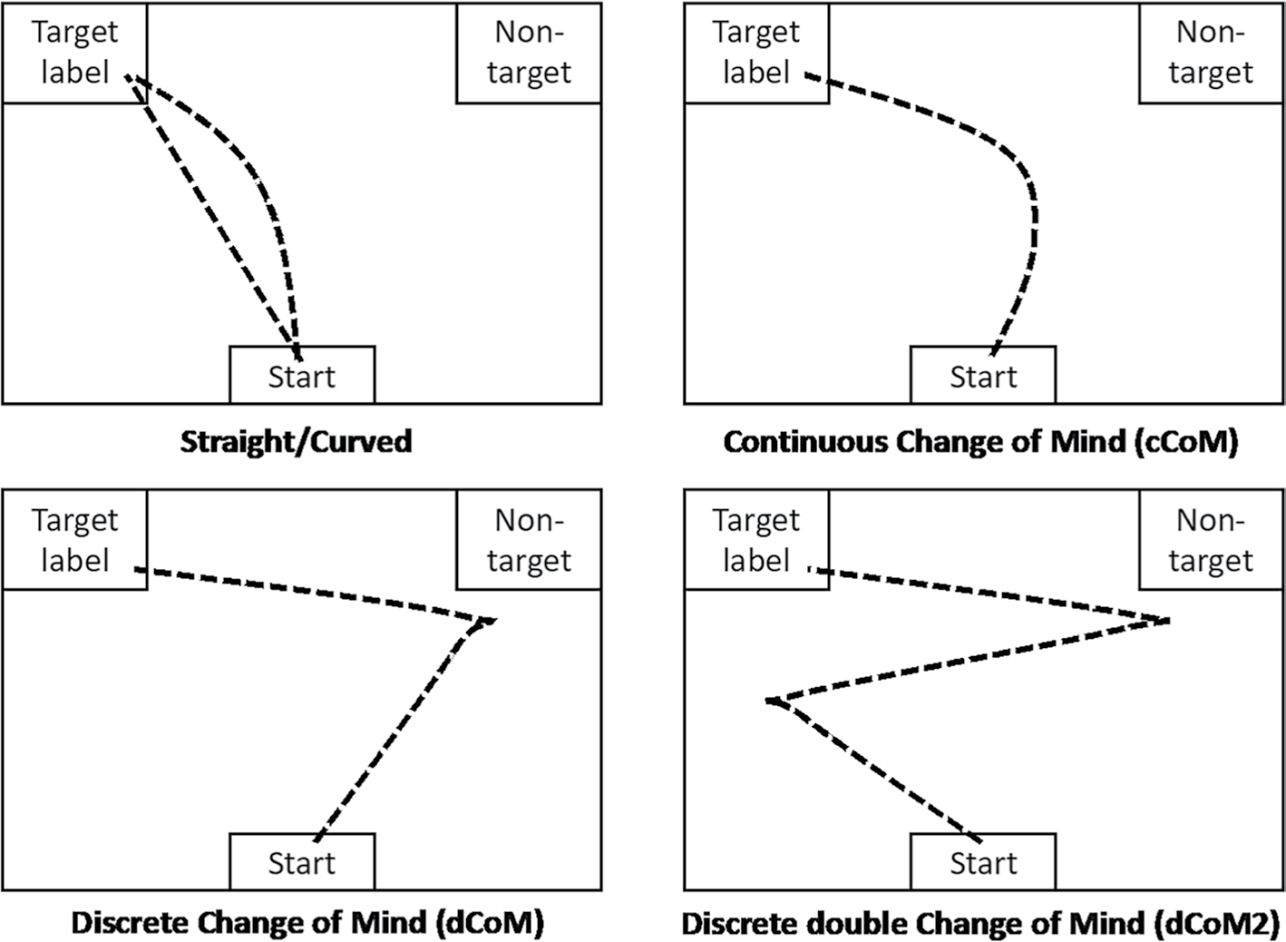
Schematic illustration of a Mouse-Tracking identification task and five trajectory prototypes

The second motivation of the present study is to explore the usefulness of Mouse Tracking (MT) technology (Farmer et al., 2007; Spivey et al., 2005; Stillman et al., 2018) in understanding cognitive competition during L2 speech perception. The MT paradigm is an identification-based paradigm in which participants move a mouse cursor from a “start” position to either a (phonological or lexical) target or a distractor located in the top-left and top-right corners of a computer screen, see Fig. 1. Participants’ mouse movements during the selection process reveal to what extent they consider the distractor, and when and how many times participants change their mind during the decision-making process. This provides a continuous measure of the degree of competition between the target and distractor, as well as a temporal measure of the process. In a classic study, Spivey et al. (2005) demonstrated the phonological cohort effect in native English listeners using an MT identification task: When listeners were instructed to identify the target word *candy*, the averaged mouse trajectories curved towards the distractor when it shared the initial segments with the target (e.g., *candle*), while the trajectories to the target were straighter when the distractor was phonologically unrelated (e.g., *jacket*).

More recently, Wulff et al. (2025) have pointed out that mouse trajectories can reveal qualitative differences in decision-making, and they argue that most mouse cursor trajectories in MT studies can be classified into one of five shape categories: Straight, Curved, continuous Change of Mind (cCoM), discrete Change of Mind (dCoM), and discrete double Change of Mind (dCoM2), indicating a range of cognitive competition levels, from minimal competition (Straight trajectories) to substantial confusion (discrete double Change of Mind; dCoM2). However, empirical evidence for this classification framework is still lacking, and the present report aims to address this gap in the L2 speech perception area.

More broadly, however, we explore MT as a low-cost psycholinguistic technique for examining online linguistic processing which does not require specialised equipment or specialist training, unlike, for example, eye-tracking, or brain-imaging (EEG, fMRI) technologies, and thus it has huge potentials for linguistics researchers. An additional special advantage of MT over other methods is that mouse trajectories are readily interpretable (Farmer et al., 2007; Spivey et al., 2005; Stillman et al., 2018), while in other paradigms the participant’s intention must be *inferred* and is not directly observable. For example, eye fixations do not necessarily indicate preference or cognitive activation (it is perfectly possible to stare blankly at a screen, for instance).

To summarise, MT identification tasks are relatively under-used and under-explored as a psycholinguistic paradigm in L2 speech perception research, and existing research using MT primarily focuses on aggregated (averaged) mouse trajectories without exploring the distribution of different trajectory types in various testing conditions (e.g., Wang & Bundgaard-Nielsen, 2023; Wang et al., 2022). This means that it is not clear how or whether an MT identification task will lead to similar results as traditional keystroke paradigms, e.g., AXB/AX discrimination tasks. Therefore, the present study addresses the following research questions:

- RQ1: Do L1 Mandarin learners of English have difficulty differentiating English /tʃ/-/tɹ/ and /dʒ/-/dɹ/ contrasts? If so, is the difficulty level modulated by the following vowel context, as indicated by loanword phonology?
- RQ2: How does an MT identification task compare to traditional key-stroke paradigms, e.g., an AXB discrimination task? Additionally, how does perceptual difficulty level affect the mouse trajectories in L2 speech perception?

## Methods

### Participants

The participants of the present study were 20, right-handed, native Mandarin speakers (*M*_*age*_ = 24.3, *SD* = 2.9, 19 females). At the time of testing, all were international students at an Australian university. The participants originally came from different Chinese provinces and had exposure to different Mandarin/Chinese dialects (the differences are irrelevant to the focus of the present study as no Chinese dialects permit stop-rhotic sequences). The participants completed their primary and secondary education in China, where English was a compulsory subject in the curriculum, and on average, they had learnt English for 13.1 years (*SD* = 3.6). On average, the participants had lived 2.2 years (*SD* = 2.9) in Australia or other English-speaking societies.

To assess the participants’ level of English, our participants completed an English vocabulary size test (Nation & Beglar, 2007), as psycholinguistic research suggests that vocabulary size is a strong indicator both of target language proficiency (Stæhr, 2008), and a strong predictor of L2 listeners’ segmental perception and production performance (Bundgaard-Nielsen et al., 2011a, 2011b, 2012). On average, the participants had an English vocabulary size of 8015 (*SD* = 1124). According to previous research, a vocabulary size of 6000–7000 words suffices unassisted reading and speaking in English (Hirsh & Nation, 1992; Nation, 2006), and therefore our participants would best be described as a group of advanced L2 users (consistent with having lived on average more than two years in an English-speaking environment for study in a tertiary institution). No participant reported any medical conditions of speech or hearing. The study was approved by the ethics committee at the researchers’ institution, and the participants gave written consent before participating in the experiment, and they received a small payment for their time.

### Stimuli

**Fig. 2:**
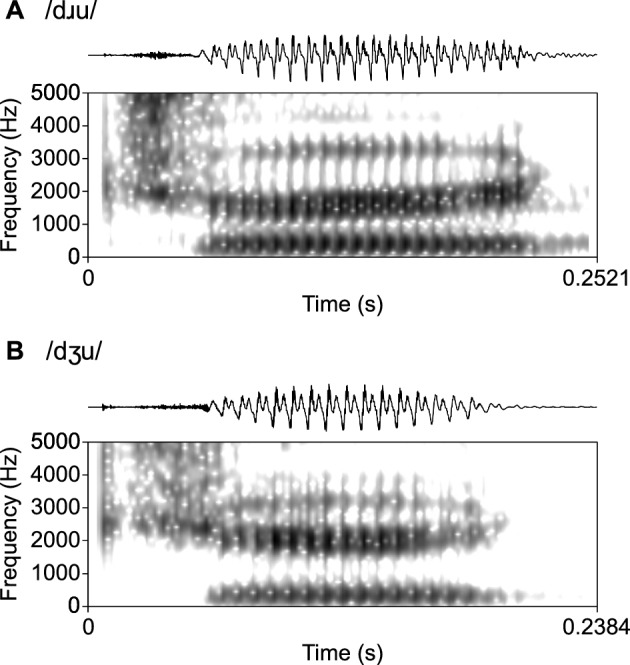
Oscillograms and spectrograms of sample stimuli produced by a male Australian English speaker. The second syllable /ti/ is not shown

The stimuli used in the present study were English disyllabic pseudowords /dʒiti, dɹiti, dʒuti, dɹuti, tʃiti, tɹiti, tʃuti, tɹuti/, produced by a male, phonetically trained speaker from Australia. The speaker articulated each nonce word using a clear citation speech style to enhance the acoustic distinctions, and all pseudowords were given a trochaic prosodic structure, such that the first syllable was stressed and the second was unstressed, consistent with English lexical stress patterns. We excluded tokens of the target nonse words with list-initial and list-final intonation pattern. We used pseudowords to reduce the influence of lexical information from the listeners’ native and second languages, with the exception that /dʒuti, tʃiti, tɹiti/ might be perceived as English real words ‘*duty*’, ‘*cheaty*’, and ‘*treaty*’. Figure 2 presents the spectrograms of two sample words.

The eight stimuli formed four critical contrasts for testing Mandarin listeners’ perception of affricate onsets: /dʒi/-/dɹi/, /dʒu/-/dɹu/, /tʃi/-/tɹi/, and /tʃu/-/tɹu/ representing a two-by-two design of both the English voicing distinction (voiced *vs* voiceless) and the effect of the following vowel (/i/ *vs* /u/). We also included /tʃu/-/dʒu/ and /dʒi/-/dʒu/ as two control conditions. The average duration of the stimuli was 621 ms (*SD* = 46), and the mean duration of the vowels in the first syllables was 156 ms (*SD* = 25). In the voiced affricate onsets, the mean voice-onset-time (VOT) was 55 ms (*SD* = 6), while in the voiceless onsets the mean VOT was 109 ms (*SD* = 9). All stimuli were auditorily checked and prepared in the phonetic software *Praat* (Boersma & Weenink, 2023).

### Procedures

The present study used a design of two perception tasks: An AXB discrimination task and a Mouse Tracking (MT) identification task. All participants completed the tasks in the same order on two consecutive days.

#### AXB Discrimination

Participants first completed an AXB discrimination task, which is a commonly used paradigm for testing auditory discrimination by sample comparison (Strange & Shafer, 2008). On each AXB discrimination trial, the participants heard a sequence of three stimuli (A, X, B) with an interstimulus interval (ISI) at 1.0 s. All four triplet types (AAB, ABB, BAA, BBA) were equally represented. In total, the AXB task had 144 trials (six contrasts, four triplets, six repetitions). The first and last stimuli were always different, and the second stimulus (X) was either phonologically identical to the first or the last stimulus (but always acoustically different). The relatively long ISI value was used to encourage phonological processing rather than lower-order phonetic processing (Werker & Tees, 1984). In the current task, the listener had 3.0 s to decide whether the first two or the last two stimuli were more similar by pressing the “*F*” key (X = A) or the “*J*” key (X = B) on their keyboard.

In addition to the four critical contrasts mentioned above (see Sect. "Stimuli"), we included two control contrasts, /tʃu/-/dʒu/ and /dʒu/-/dʒi/. Including the /tʃu/-/dʒu/ contrast allowed us to check whether the Mandarin-speaking participants had difficulty perceiving the voicing distinction, while the /dʒu/-/dʒi/ contrast allowed us to check whether the participants had difficulties with the /i/-/u/ vowel contrast. The trials were developed and delivered in a randomised order using *PsyToolkit* (Stoet, 2010, 2017). Due to COVID-19 pandemic restrictions on social gatherings and research on the university campus at the time of data collection, all participants completed the task in a quiet room in their homes.

#### MT Identification

The second task was an identification task using Mouse Tracking (MT) technology developed using *PsychoPy* (Peirce, 2007). During this task, each participants’ screen was normalised to a 2 units-by-2 units canvas. Following the conventional MT paradigm, a “start” box was cantered at the bottom of the screen [0, 0], while the two response labels were presented at the top-left [− 1, 2] and top-right [1, 2] corners of the screen. On each trial, the listener first clicked the “start” box to play the auditory stimulus, and then selected one of two category labels (“CH” vs “TR” representing /tʃ/ vs /tɹ /or “J” vs “DR”, representing /dʒ/ vs /dɹ/) presented on the screen. Participants were instructed to move the mouse as quickly as possible, but we did not give trial-by-trial feedback about their initiation time. The cursor trajectories were recorded during the response procedure. When perception is easy, we expect that the MT trajectory should resemble a straight line connecting the “start” button and the correct response (left or right corner), while perceptual conflicts will be expected to result in curved or in zig-zag trajectories. The sample rate of the MT trajectory recording was 60 frames per second (FPS) for all participants, i.e., two adjacent mouse locations represent the magnitude of cursor displacement in a 16.7 ms period. Participants completed the task using a self-prepared computer mouse (touchpads were not allowed). Because participants completed the task in potentially different testing environments, analyses were restricted to within-participant comparisons, i.e., paired performance metrics across conditions. Between-participant comparisons were avoided, as they could be more susceptible to variability arising from system-specific delays (e.g., latency differences between wired and Bluetooth mouses).

After a trial, the “start” box was presented again and the participants needed to click on the box to activate the next trial. This procedure ensured that the mouse cursor started from approximately the same location for each trial. The stimuli were presented in a randomised order, while the directions (top-left *vs* top-right) of correct responses were counterbalanced. The task had 288 trials (four consonants, two vowels, three tokens per combination, two directions, six repetitions). For the purposes of data analysis, all rightward trajectories were mirrored as leftward trajectories, such that the top-left corner always presented the correct label, and the top-right corner always presented the incorrect non-target/distractor. Following this, we first calculated the accuracy of the participants’ identification as an end-point measure. We also classified each of the MT trajectories as one of the five prototype groups: Straight, Curved, cCoM, dCoM, and dCoM2 (Wulff et al., 2025) using the “mousetrap” software package (Kieslich, 2021) which assesses the spatiotemporal complexity of the MT trajectories via two metrics, including the total trajectory distance (in normalised unit), and the response time (RT, in second). The data and processing scripts are available in the OSF folder at https://osf.io/cw3hp/.

## Results

### AXB Discrimination Results

The mean accuracy data from the AXB discrimination task are summarised in Table 1. For the two control contrasts, L1 Mandarin speakers achieved very high accuracy measures for /tʃu/-/dʒu/ (95.2%) and /dʒi/-/dʒu/ (97.0%), indicating that the participants did not have difficulty perceiving the English VOT contrast nor the English vowel contrast when the phonological context is controlled (or difficulty understanding and executing the task). These two pairs were not included in the analysis of critical trials, as reported below.

**Table 1:** AXB discrimination accuracy of English affricate onsets by Mandarin listeners

Contrast	Condition	Accuracy (%)	*SD*
/dʒ/-/dɹ/	/u/	95.6	6.0
	/i/	96.7	8.6
/tʃ/-/tɹ/	/u/	88.5	13.4
	/i/	97.9	4.0
/tʃu/-/dʒu/	Control: Voicing	95.2	9.0
/dʒi/-/dʒu/	Control: Vowel	97.0	6.9

In the four critical conditions, ceiling-level accuracy was observed for /dʒu/-/dɹu/ (95.6%), /dʒi/-/dɹi/ (96.7%), and /tʃi/-/tɹi/ (97.9%), while /tʃu/-/tɹu/ had a mean accuracy of 88.5%. To analyse the differences, we built a generalised linear mixed-effects model for the four critical conditions (GLMM, binomial link; fixed effect: Contrast; random effect: Intercepts for participants only since inclusion of random slopes led to over-fitting). The model was then checked by a Wald Chi-squared test, which revealed a significant effect of contrast, *χ*^*2*^(3) = 50.4, *p* < .0001. We then carried out a series of post hoc tests based on estimated marginal means (EMMs), which showed that the accuracy of /tʃu/-/tɹu/ was lower than that in the other three contrasts (*p* < .0001 for three comparisons), while the differences between the other three pairs were non-significant (*p* > .05 for three comparisons). In summary, L1 Mandarin listeners demonstrated some difficulty perceiving the /tʃu/-/tɹu/ (but not /tʃi/-/tɹi/) in the AXB task.

### MT Identification Results

We excluded one participant from the MT analyses as they failed to understand the test instructions. The averaged MT identification accuracy data from the remaining 19 participants is summarised in Table 2. Consistent with our predictions, and with the results from the AXB discrimination task, the L1 Mandarin-speakers were highly accurate in their identification of affricate onset pairs when these followed an /i/ vowel (Mean accuracy > 95%). Preceding /u/, the participants accurately identified the affricates 68.2% (for /tʃu/-/tɹu/), and 75.6% of the time (for /dʒu/-/dɹu/). Here, the accuracy rates in the two difficult conditions showed clear perceptual difficulty when participants had to interpret the perceived segments using phonological labels. Note that in AXB discrimination, the participants still achieved relatively good accuracy when multiple contrastive segments were presented in the same trial, suggesting a potential paradigmatic difference: Mandarin speakers found it more challenging to identify English affricates in isolated words using orthographical-phonological labels than to perceptually differentiate contrastive onsets in an auditory AXB task.

**Table 2:** Identification accuracy of English affricate onsets by Mandarin listeners

Contrast	Condition	Accuracy (%)	*SD*
/dʒ/-/dɹ/	/u/	75.6	16.1
	/i/	95.5	10.6
/tʃ/-/tɹ/	/u/	68.2	16.2
	/i/	96.8	4.5

The response data were again modelled in a generalised linear mixed-effects model (GLMM; fixed effects: Onset contrast, vowel condition; random effects: Intercepts for participants), and then checked by a Wald Chi-squared test, which revealed a significant main effect of vowel condition, *χ*^*2*^(1) = 453.8, *p* < .0001, a significant effect of onset, *χ*^*2*^(1) = 11.6, *p* = .0007, as well as a significant interaction effect between the vowel condition and the onset contrast, *χ*^*2*^(1) = 12.2, *p* = .0005. Therefore, we carried out a series of six post hoc tests based on estimated marginal means (EMMs), which revealed that the /i/-condition led to higher accuracy measures than the /u/-condition for both onsets (*p* < .0001 for four comparisons). Additionally, the accuracy of /tʃu/-/tɹu/ was lower than /dʒu/-/dɹu/ (*p* < .0001), but the accuracy difference between /tʃi/-/tɹi/ and /dʒi/-/dɹi/ was not significant (*p* = .2747) after *p*-value adjustment (Tukey’s method). To summarise, for accuracy, {/tʃi/-/tɹi/, /dʒi/-/dɹi/} > /dʒu/-/dɹu/ > /tʃu/-/tɹu/.

**Fig. 3:**
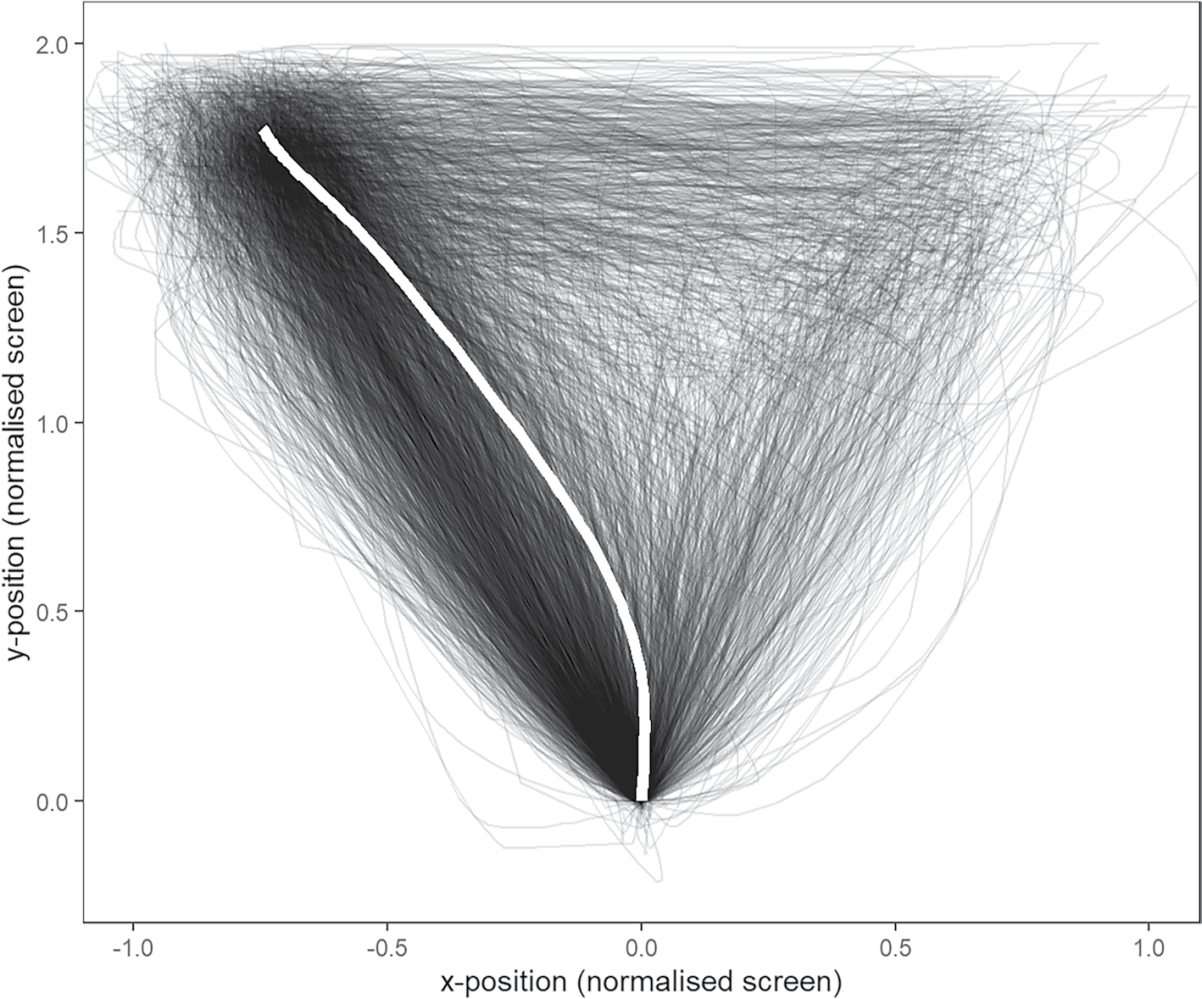
MT recordings after spatial normalisation and the aggregated mouse trajectory (white line)

In addition to response accuracy, the MT identification task recorded the hand movements into mouse cursor trajectories when performing the identification responses. This measure provided access to valuable information about the participants online perceptual processing during the task. Figure 3 above visualises the mouse trajectories in all correctly responded trials, and the white line indicates the aggregated/mean trajectory in all valid trials.

We first inspected all 5472 individual trajectories and removed any off-track recordings, leaving 4114 successfully executed trajectories for analysis. Next, we conducted further data cleaning procedures based on the suggestions in Wulff et al. (2025): We first removed incorrected responses (*N* = 570) and trials with an abnormal frame rate (*N* = 18).

Since participants could freely move their mouse cursors during the task, we also observed some over-complex trajectories which could not be easily classified into one of the five prototypes. To control these outliers, we removed all trials with *z* score of total distance above 1.96 (*N* = 165), which is analogous to the concept of an alpha level at 0.05 in hypothesis testing. Next, we also inspected the distribution of RT in the remaining trials, and 99% of the trials had an RT below 4.5 s, so we further removed the top 1% of long responses (*N* = 39). Finally, since we use mouse trajectories to infer online process, trials with a very long initiation time were less reliable because participants could had made decisions before moving their mind, and then the trajectories were no longer indicators of their concurrent phonological processing. Previous studies have used different cutoff points from 300 to 750 ms (Wulff et al., 2025). We used a bottom-up approach to determine the cutoff point based on the percentiles: 80% of the trials had an initiation time under 750 ms, but only 34% of the trials had an initiation time below 300 ms. To maximally use the available data, we adopted the upper boundary at 750 ms and removed 664 trials.

**Fig. 4:**
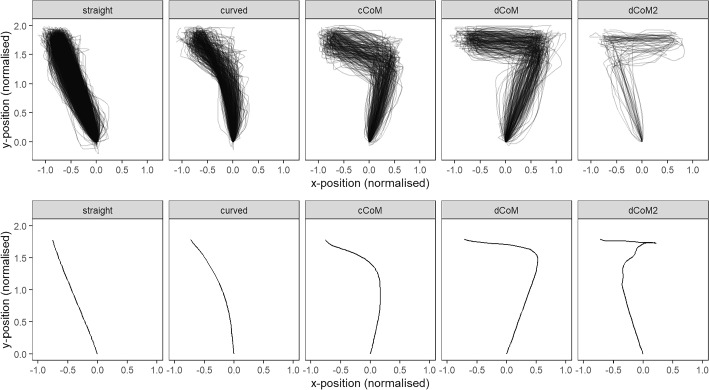
MT recordings and aggregated trajectories in each classified prototype group

After these data cleaning procedures, the remaining MT trajectories (*N* = 2575) were classified into one of five prototypes using the “mousetrap” software package (Kieslich, 2021): Straight, Curved, Continuous Change of Mind (cCoM), discrete Change of Mind (dCoM), and discrete double Change of Mind (dCoM2), see Fig. 4.

**Fig. 5:**
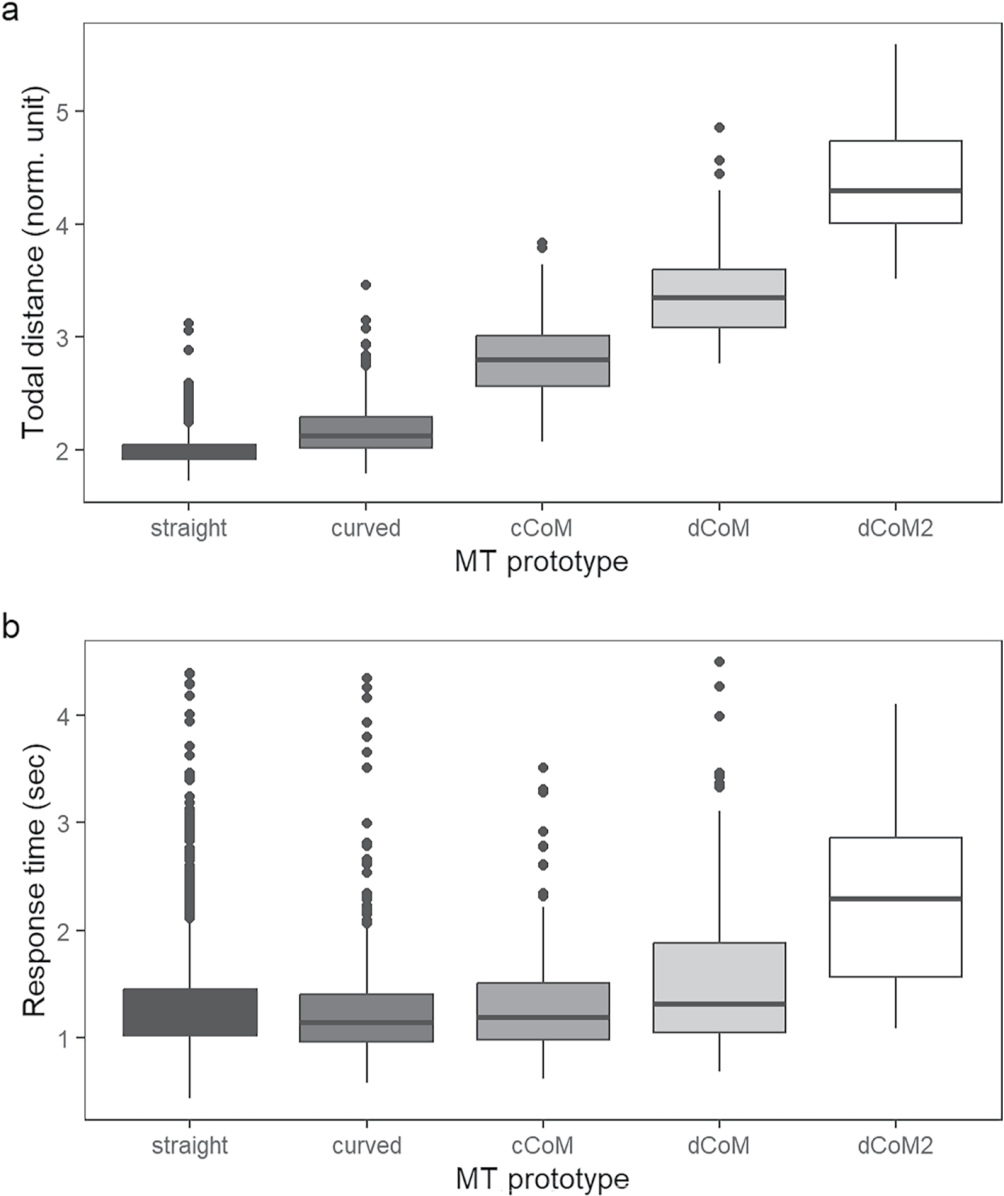
Distribution of total trajectory distance and response time in each prototype

As the first step of the analysis, we explored the spatiotemporal properties of the cursor trajectories, and in particular we compared the total trajectory distance and RT in each prototype category across all contrast conditions, see Fig. 5. For inferential statistics, we built linear mixed-effects models (LMMs; fixed effect: Trajectory prototype; random effects: Intercepts for participants, since inclusion of slopes led to overfitting). For total distance, there was a significant effect of prototype, *χ*^*2*^(1) = 12037, *p* < .0001, and all pairwise comparisons were significant in the post hoc tests, such that Straight < Curved < cCoM < dCoM < dCoM2 (*p* < .0001 for all comparisons). For RT, we first performed a logarithmic transformation to contract skewness (Whelan, 2008), and then fitted another LMM, which also showed a significant effect of prototype, *χ*^*2*^(1) = 227.24, *p* < .0001. In the post hoc tests, the only non-significant comparison was between the two easy trajectory prototypes, Straight and Curved trajectories (*p* = .6757, Tukey-adjusted), and between Curved and cCoM trajectories (*p* = .1057, Tukey-adjusted), while all the other comparisons were significant (*p* < .0014 for eight comparisons). This finding showed that while Curved trajectories were generally longer than Straight trajectories, responses with these two trajectory shapes were executed at a similar rate, and therefore the differences in cognitive competition between these two categories might not be as straight-forward as suggested by the total distance metric. To summarise, the RT data showed a pattern that {Straight, Curved} ≤ cCoM < dCoM < dCoM2. Taken together, the prototype clustering analysis has successfully classified the MT trajectories into five categories with different levels of spatiotemporal complexity.

**Fig. 6:**
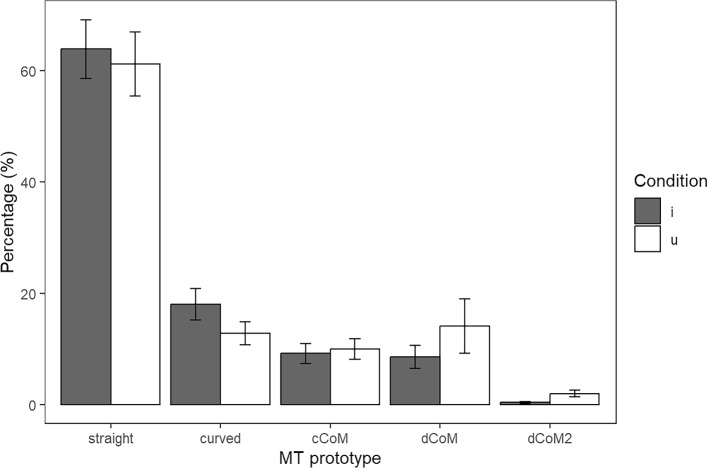
Proportion of trajectory prototypes in each condition. Error bars indicate standard errors of the mean

Having established that the five prototype categories did differ significantly in terms of total trajectory distance and RT (with the single exception of Straight vs Curved in terms of RT), we were particularly interested in the distribution of trajectory categories in the easy /i/ context versus difficult /u/ context, see Fig. 6. In the visualisation, we did not distinguish the voiced and voiceless onsets due to their similarity in identification results, although the voiceless contrasts were slightly more challenging than the voiced contrasts. Descriptive statistics showed that the /i/ context had a higher proportion of Straight lines than the /u/ context (63.8% vs 61.2%), as well as more Curved trajectories (18.0% vs 12.8%). On the other hand, the /u/ context had more cCoM trajectories than the /i/ context (10.0% vs 9.2%), as well as dCoM trajectories (14.1% vs 8.6%), and dCoM2 trajectories (2.0% vs 0.4%). The pattern emerged from these data was that the /u/ contexts had more curves indicating changes of mind.

To simplify the analysis, we collapsed the five prototypes into one binary variable, Change of Mind (CoM), which is zero when the line was straight or curved, but one when it was one of cCoM, dCoM, dCoM2. To analyse the frequency of mind-changing, we build a GLMM (Binomial link; fixed effects: Vowel, onset contrast; Random effect: Intercepts for participants, since inclusion of slopes led to overfitting), which revealed a main effect of vowel context (/i/ vs /u/), *χ*^*2*^(1) = 11.73, *p* = .0006, a main effect of onset contrast (/dʒ/-/dɹ/ vs /tʃ/-/tɹ/), *χ*^*2*^(1) = 6.51, *p* = .0107, while the interaction was not significant. We then carried out two post hoc tests, which confirmed that the /u/ context, as well as the voiced onsets, led to more changes of mind.

## General Discussion

The present study examined the discriminability of English affricate and stop-rhotic sequences using an AXB discrimination and an identification task using MT technology. The results suggest that the perception of affricate consonants can be challenging for L2 speakers whose native phonology does not have phonetically and phonotactically equivalent segments. The accuracy results from both the AXB discrimination task and the MT identification task showed that the Mandarin speakers’ perception of English affricate onsets was influenced by the following vowel contexts, i.e., differentiating /tʃ/-/tɹ/ and /dʒ/-/dɹ/ contrasts was easy in the /i/ context, but substantially more difficult in the /u/ context. This contextual effect might be rooted in the constraints of Mandarin syllable phonotactics: Mandarin listeners can potentially exploit the labial rounding gesture in /tɹ/ and /dɹ/ (i.e., /tɹ, dɹ/ → /tʃw, dʒw/) but such exploitation is not available when the labial gesture is masked by a following rounded /u/ vowel. When the detection of gestural differences becomes difficult, Mandarin listeners experience difficulty in differentiating these two pairs of English onsets. We acknowledge that AXB discrimination and MT identification do not provide *direct* evidence of the proposed repairing strategy, and we cannot rule out other possibilities to explain this phenomenon. Future research should use other tasks to directly assess cross-language adaptation (e.g., transcribing L2 sounds using L1 phones) or perceptual similarity (e.g., rating scores of similarities between L2 sounds and L1 categories). At the same time, our data suggest that the voiceless contrast tends to be more challenging than the voiced contrast, as revealed in both AXB and MT identification tasks, which is consistent with some previous reports, e.g., English listeners find it more challenging to discriminate Hebrew /tl/-/kl/ contrast as compared to /dl/-/gl/ (Best & Hallé, 2010; Hallé & Best, 2007). This finding indicates that the duration of aspiration might influence the gestural salience of the neighbouring segments: The following segments might be masked by the glottal airflow in voiceless plosive consonants. Future studies could also explore how voicing differences affect acoustic cues related to the rhotics and the following vowels to further elucidate the exact nature of this nuanced but significant effect.

As a methodological contribution, this paper presents a first systematic analysis of the distribution of different mouse cursor trajectories in L2 speech perception using a recently-developed classification framework (Wulff et al., 2025), while most previous research focuses on the analysis of aggregated mouse trajectories without exploring the *qualitative* differences among mouse trajectories classified based on prototype shapes (Wang & Bundgaard-Nielsen, 2023; Wang et al., 2022). Our analysis suggests that different trajectory types (Straight, Curved, cCoM, dCoM, and dCoM2) are associated with different levels of spatiotemporal complexity, in terms of the total trajectory distance and the time for executing such responses. Straight and Curved trajectories indicate decision-making processes that involve minimal category competition and thus can be more often observed in an easy condition (e.g., the /i/ context). Various kinds of Change of Mind (CoM) trajectories indicate substantial competition and perceptual confusion, and they can be more often observed in difficult conditions (e.g., the /u/ context). More importantly, MT identification offers readily interpretable visualisations of the L2 learners’ online process of speech stimuli, which is an extension of traditional keystroke identification paradigm. However, a future study should carry out the MT task in a more controlled laboratory setting, because using self-prepared equipment may lead to unexpected individual variations due to different test settings, for instance, one limitation of the present study is that a number of mouse trajectories were off-track and overall only around 75% of the mouse recordings were accurately logged (and some participant were more affected than others). It is of course also possible that the discarded over-complex trajectories that do not fit into the five-prototype categorisation system may still reflect some language processing scenarios, as it is possible for people to change their mind three times or even more.

These caveats regarding the study interpretation aside, the present study demonstrates that MT offers a low-cost—additional or complementary—experimental paradigm that can be effectively used in cross-language speech perception, and that MT decision trajectories can offer a unique window into participants’ decision-making process including the changes of mind especially when they experience phonological interference. That being said, it is worth noting that MT is a paradigm that piggybacks on a traditional identification task, and it is still a measure of overt motor responses (similar to key-pressing) rather than directly measuring attention allocation as in an eye-tracking task.

Nevertheless, in the present study we did not directly test Mandarin listeners’ online adaptation or perceptual assimilation of English affricate-vowel phonemic sequences. Some recent studies showed that the perceptual difficulty level of nonnative sequences can be predicted from the phonological mapping of such sequence categories between the L2 and the listener’s L1 (e.g., Kilpatrick et al., 2019; Wang et al., 2023a, 2023b). A further study, therefore, is warranted to explore how such sequences lead to cross-linguistic integrations between the listeners’ native and non-native phonological systems. In addition, we observed that the accuracy measures of the critical contrasts in the AXB were generally higher than those in the MT identification task (especially, /dʒu/-/dɹu/), suggesting a paradigmatic difference: Some phonological contrasts can be more difficult to differentiate when presented individually (identification), but perception might be easier if both categories are presented for direct comparison (discrimination). This finding suggests that AXB and identification tasks may tap into different modes of speech perception, and thus their results share both similarities and complementarities.

Another potential confounding factor in the present study was the relative frequency of affricate-vowel sequences and their neighbourhood density, as psycholinguistic research has shown that listeners tend to have difficulty perceiving phonemic sequences with a low frequency (e.g., /ðʌʃ, θɛg/) or a large phonological neighbourhood (e.g., *cat* might be misheard as *cap* or *can*) (Luce & Pisoni, 1998; Vitevitch & Luce, 1998; Vitevitch et al., 1999). In the current study, all four critical affricate-vowel sequences are common /dʒi, dʒu, tʃi, tʃu/ in English, but they still have different absolute frequency values. To further tease out the frequency and neighbourhood effects, future studies could test a range of different consonant–vowel sequences varying frequency measures and neighbourhood sizes and then add these factors as covariates. Relatedly, since the participants in the present study were L2 English learners, it is difficult to predict their sensitivity to frequency and neighbourhood density without control groups, including native speakers and naïve listeners. For instance, native speakers should have no difficult discriminating the pairs or identifying the individual phones, provided that all sequences were commonly found in English words. Naïve listeners or less experienced L2 learners should have difficulty in identification as well as in discrimination. A future study can recruit participants with more diverse language backgrounds and explore how target language experience and proficiency may moderate affricate perception in Mandarin listeners.

## Data Availability

The datasets generated by the experimental research and the analysis steps of the current study are documented in Supplementary Materials, available on OSF database, https://osf.io/cw3hp/.

## Appendix 1

Outlier trajectories excluded for trajectory analysis.
